# Ventricular Asystole During Le Fort I Orthognathic Surgery: A Case Consistent with Trigeminocardiac Reflex and a Mini Review

**DOI:** 10.3390/clinpract16010013

**Published:** 2026-01-07

**Authors:** Sucharu Ghosh, Sandra Armanious, Anirudh Nair, Zeynep Ulku, Daniel Sultan, Robert Pellecchia

**Affiliations:** Department of Oral and Maxillofacial Surgery, NYCHHC/Lincoln Medical Center, 234 E 149th St, Bronx, NY 10451, USA; armanios@nychhc.org (S.A.); naira7@nychhc.org (A.N.); ulkuz@nychhc.org (Z.U.); sultand2@nychhc.org (D.S.); robert.pellecchia@nychhc.org (R.P.)

**Keywords:** trigeminocardiac reflex, nasocardiac reflex, Le Fort I osteotomy, orthognathic surgery, asystole, lateral nasal osteotomy, rhinocardiac reflex, case report

## Abstract

Introduction: The trigeminocardiac reflex (TCR) is a brainstem reflex in which trigeminal stimulation precipitates abrupt vagally mediated cardiovascular changes, ranging from bradycardia to asystole. While classically described during down-fracture or pterygomaxillary disjunction in Le Fort I osteotomy, rhinocardiac events from lateral nasal wall manipulation are less emphasized in orthognathic surgery. Case presentation: A 32-year-old man undergoing Le Fort I osteotomy developed ventricular asystole during lateral nasal osteotomy. The maneuver was stopped immediately; chest compressions and a single dose of epinephrine were administered, with return of spontaneous circulation within approximately one minute. Surgery was aborted and the patient was transferred to the surgical ICU. Clinical discussion: The temporal association with lateral nasal wall manipulation, in the setting of controlled hypotension and multimodal anesthesia, is most compatible with a peripheral (V2) TCR-type event, although drug-related and hemodynamic contributors cannot be excluded. A mini review of orthognathic TCR reports underscores recurring high-risk steps (down-fracture, pterygomaxillary disjunction, mandibular maneuvers) and highlights lateral nasal osteotomy as a potential additional trigger. Management principles remain the immediate cessation of the stimulus, optimization of oxygenation and ventilation, anticholinergics for bradycardia, and epinephrine/advanced cardiac life support for instability or arrest. Conclusion: Lateral nasal osteotomy may trigger a TCR-like event with severe bradyarrhythmia or asystole during Le Fort I osteotomy, particularly in hemodynamically vulnerable patients. Anticipation, swift recognition, and prompt, protocolized management are essential for favorable outcomes.

## 1. Introduction

The trigeminocardiac reflex (TCR) is an evolutionarily conserved brainstem reflex that produces abrupt changes in heart rate, blood pressure, and respiration following stimulation of the trigeminal nerve [1]. Bernard Aschner first described the ocular variant of TCR in 1908, termed the oculocardiac reflex [2]. Subsequent reports demonstrated that stimulation of any trigeminal division can evoke similar responses, hence Shelly and Church suggested the broader term trigeminocardiac reflex [3]. The afferent pathway includes peripheral fibers of the trigeminal nerve projecting via the Gasserian ganglion to the trigeminal sensory nucleus. Short internuncial fibers within the reticular formation connect this nucleus to the vagal motor nucleus, forming an efferent pathway that travels via the vagus nerve to the myocardium, resulting in bradycardia and negative inotropy [4]. When exaggerated, the reflex may result in profound hypotension or ventricular asystole.

Although TCR is commonly associated with neurosurgical and ophthalmic procedures, it can occur during maxillofacial surgeries that manipulate the maxillary or mandibular branches of the trigeminal nerve [5,6]. Le Fort I osteotomy involves separation and mobilization of the maxilla at the level of the pterygomaxillary suture and posterior maxillary wall; these manipulations can directly stimulate the palatine and superior alveolar nerves, making the procedure susceptible to TCR [7]. Nasal manipulation can elicit a related nasocardiac (rhinocardiac) reflex, with bradycardia and even arrest reported during rhinologic procedures, supporting a V2-mediated mechanism relevant to lateral nasal osteotomy [8].

To our knowledge, after a structured search of the orthognathic literature, ventricular asystole temporally associated with lateral nasal osteotomy has not been previously described; most orthognathic TCR cases cluster around down-fracture, mobilization, or pterygomaxillary disjunction. This case therefore expands the list of high-risk steps during Le Fort I by drawing attention to a possible nasocardiac trigger at the lateral nasal wall. We also use this case to review diagnostic criteria, discuss anesthetic and pharmacologic cofactors, and summarize practical implications for pre-briefing, monitoring, and rapid response. This case report has been reported in line with the SCARE criteria [9].

## 2. Case Presentation

A 32-year-old African American man was referred for correction of dentofacial deformity (maxillary hypoplasia). He denied past medical history except anxiety and depression. No known drug allergies; not on chronic medications. He had no known surgical history. Family history was noncontributory. He denied substance use except for occasional marijuana use. He was scheduled for Le Fort I osteotomy.

*Anesthesia and setup.* The patient underwent general anesthesia with standard monitoring and nasotracheal intubation. Local anesthetic with epinephrine was infiltrated at the start of the procedure. Anesthesia was induced with propofol 150 mg IV, fentanyl 100 µg IV, midazolam 2 mg IV, and rocuronium 50 mg IV. See Table 1 for detailed timeline of events.

*Index event.* During the lateral nasal osteotomy, the patient developed ventricular asystole. The maneuver was immediately stopped; chest compressions were initiated, and epinephrine (single dose) was administered, with return of spontaneous circulation documented within approximately one minute. Based on the anesthesia record and code documentation, the exact duration of electrical asystole cannot be determined more precisely. The case was aborted, and the patient was transferred to the surgical intensive care unit (SICU) for monitoring.

*Post-event evaluation.* The ECG showed sinus bradycardia, otherwise normal, with no ischemic changes. High-sensitivity troponin (ng/L) increased from 157 (11:28) to 702 (16:15) and 706 (19:20), then down-trended to 531 (22:43). Transthoracic echocardiography and CT pulmonary angiography were normal. Electrolytes: Na 140 mmol/L and K 3.9 mmol/L. An arterial blood gas obtained after the event showed pCO_2_ 47.2 mmHg, pO_2_ 230 mmHg, and lactate 2.93 mmol/L. ICU telemetry revealed no significant recurrent arrhythmias. The pattern of a moderate troponin rise with subsequent decline, in the absence of imaging evidence of structural disease, was considered most consistent with peri-arrest myocardial injury and catecholamine effect, although a contribution from transient supply–demand mismatch could not be excluded.

*Hospital course.* Postoperatively, the patient remained intubated and sedated in the surgical ICU and underwent routine ventilator weaning with spontaneous breathing trials. He was extubated once he met standard respiratory and hemodynamic criteria and was awake, alert, and oriented, moving all extremities without focal neurologic deficit. The Foley catheter was removed with a successful trial of void. He tolerated a full liquid diet and ambulated with adequate pain control on oral medications. No further arrhythmic episodes occurred on telemetry, and he was discharged home on postoperative day 2. No re-attempt at osteotomy is planned currently.

*Differential diagnosis.* The temporal linkage to lateral nasal osteotomy strongly implicates TCR via maxillary (V2) afferents. Similar vagally mediated events are described with nasal disinfection, injections, packing, and endoscopy, including cardiac arrest in extreme cases [8]. However, additional mechanisms likely contributed. The episode occurred during controlled hypotension after administration of labetalol and hydralazine, with ongoing opioid and propofol dosing, which would all be expected to blunt sympathetic responses. The moderate troponin rise with subsequent down-trend, normal echocardiography and CT pulmonary angiography, and absence of recurrent arrhythmias on telemetry are compatible with peri-arrest myocardial injury and catecholamine effect rather than primary structural heart disease, but occult ischemia or an underlying arrhythmogenic substrate cannot be completely excluded. A purely drug-mediated vagotonic or cardioinhibitory event also remains possible. Taken together, the event is best interpreted as a probable TCR-type reflex occurring in the context of multifactorial anesthetic and hemodynamic vulnerability rather than an isolated, pharmacologically neutral reflex.

## 3. Literature Review and Discussion

Stimulation of the trigeminal nerve activates an afferent arc that transmits impulses through the Gasserian ganglion to the trigeminal sensory nucleus. Short internuncial fibers then connect to the motor nucleus of the vagus nerve, sending pre-ganglionic parasympathetic efferent fibers to the heart [4]. Activation of the vagal efferent produces negative chronotropic and inotropic effects, leading to bradycardia, hypotension, apnea, and, in severe cases, asystole.

### 3.1. Epidemiology

The reported incidence of TCR varies widely from 5% to 90% depending on the surgical site and stimulus [10,11]. In craniofacial surgery, the overall incidence is about 20%, with orthognathic procedures having an incidence around 9% [12]. Facial trauma surgeries carry the highest risk (up to 32%), whereas lower-face reconstruction has the lowest risk (3.6%) [12]. A 2025 review of TCR in orthognathic surgery identified ten cases reported between 1989 and 2024; seven involved asystole and three involved bradycardia only [7]. Most episodes occurred during Le Fort I osteotomies, especially during pterygomaxillary disjunction or maxillary down-fracture. Only three cases were associated with bilateral sagittal split osteotomy (BSSO). In nearly all cases, stopping the surgical maneuver led to prompt recovery; anticholinergic drugs (atropine or glycopyrrolate) were used in six cases. Available reports do not demonstrate any clear differences in TCR incidence by race or ethnicity; therefore, patient race is reported in our case only descriptively and not as a presumed risk factor.

### 3.2. Diagnostic Criteria

*Working definition.* Classically, TCR is defined by a sudden ≥20% fall in heart rate (HR) and mean arterial blood pressure (MABP) relative to baseline, provoked by physical/electrical/chemical stimulation anywhere along the trigeminal pathway; apnea and gastric hypermotility may accompany the cardiovascular changes [13]. Some series have used a 10% threshold [14], while others broadened this definition to include any perturbations—and even paradoxical increases—in cardiovascular parameters, particularly when the reflex is initiated outside the classic central pathway [15,16].

*Cause–effect framework.* Beyond the numerical drop, diagnosis should demonstrate a cause–effect relationship between trigeminal stimulation and the autonomic response. Meuwly et al. [1,17] proposed two major and two minor criteria for clinical/research use:

Major criteria (both should be present)

1. Plausibility: a clear temporal and anatomical link—the response follows ≤5 s after stimulation of a trigeminal branch (or Gasserian ganglion/brainstem nuclei), and alternative causes (e.g., pain response) are excluded.
2. Reversibility: abolition of the stimulus leads to resolution of the hemodynamic/autonomic changes (recognizing rare “point-of-no-return” reports where asystole persists despite cessation [18].

Minor criteria (supportive, not required)

1. Repetition: the phenomenon reappears with repeated stimulation (ethical/practical limits often preclude testing).
2. Prevention: attenuation or absence of the response with gentler manipulation, local nerve block, or anticholinergic premedication (these are not absolute and may fail).

In our patient, anatomical and temporal plausibility was high: ventricular asystole occurred during manipulation of the lateral nasal wall in the V2 territory, in close succession to the osteotomy. However, the exact second-by-second interval between the stimulus and hemodynamic collapse was not captured, and concurrent factors such as controlled hypotension, β-blockade, vasodilators, opioids, and intermittent propofol boluses may have lowered the threshold for a vagal event. Reversibility was achieved after cessation of the maneuver together with chest compressions and a single dose of epinephrine; thus, recovery did not occur with stimulus removal alone but is compatible with “point-of-no-return” TCR descriptions in which arrest persists despite withdrawal of the trigger [18]. Minor criteria could not be assessed: the maneuver was not repeated, and no specific anticholinergic premedication was used in this case. Overall, the episode fulfills many, but not all, components of a peripheral TCR according to Meuwly et al. and is best characterized as a probable V2-mediated TCR occurring in a multifactorial anesthetic context.

### 3.3. Classification

Meuwly et al. proposed an extended classification for comprehensive understanding of the TCR [17].

*Central TCR.* Elicited by manipulation of the intracranial segment of the trigeminal system—from the Gasserian ganglion to the brainstem nuclei—typically during skull base or posterior fossa procedures. Schaller et al. first characterized this phenomenon in 1999 [13]. Central TCR often manifests with abrupt bradyarrhythmia/asystole and hypotension when the brainstem reflex arc is directly engaged.

*Peripheral TCR.* Elicited by stimulation of the extrinsic (peripheral) branches of the trigeminal nerve (V1–V3). Bradycardia is characteristic; effects on blood pressure are variable because of concurrent sympathetic activation. The peripheral TCR has two subtypes: (1) the oculocardiac reflex (OCR) and (2) the maxillo-mandibular cardiac reflex [11,12,19]. Classic oculocardiac reflex arises with orbital/eyeball manipulation via V1; analogous responses can arise from V2/V3 and include nasocardiac events during lateral nasal wall/piriform rim work. These entities are all encompassed within the broader TCR construct.

Other proposed variants (e.g., diving reflex and chronic/delayed presentations) are described in the broader TCR literature.

### 3.4. Orthognathic Triggers and Risk Factors

Mechanical stretch or pressure is the principal trigger of TCR. Asystole during Le Fort I has been reported at down-fracture and mobilization [20,21]. Sudden disjunction of the pterygomaxillary suture, placement of retractors or bite blocks, and traction on the inferior alveolar or auriculotemporal nerves have all precipitated asystole [7,22]. Bilateral stimulation produces a stronger response than unilateral stimulation [19]. Other triggers include thermal or electrical stimulation and local infiltration of anesthetics or vasoconstrictors [10,12,19]. Predisposing factors include young age, male sex, high sympathetic tone, hypoxemia, hypercarbia, light anesthesia, neuromuscular blockers, opioids, β-blockers, and the strength and duration of the stimulus [5,10,12,17,19,23,24]. Controlled hypotension and use of vasoactive drugs can also contribute. Our case adds lateral nasal osteotomy as the inciting step, aligning with the ENT literature on the nasocardiac reflex from lateral nasal wall/columellar manipulation [8]. In our patient, manipulation of the maxillary segment during osteotomy—on a background of controlled hypotension and opioid/anesthetic administration—likely lowered the threshold for a TCR-type response. An arterial blood gas obtained after the event showed mild hypercapnia (pCO_2_ 47 mmHg), which may have further facilitated vagal excitability [25].

### 3.5. Anesthetic Considerations in This Case

Several anesthetic and pharmacologic factors likely contributed to hemodynamic vulnerability at the time of the event. Labetalol administered earlier in the procedure, followed by hydralazine in the period leading up to the lateral nasal osteotomy, would be expected to blunt sympathetic responses and lower systemic vascular resistance. Repeated boluses of fentanyl and intermittent propofol dosing may have further reduced sympathetic tone. Redosing of rocuronium removed somatic movement as an early warning sign of light anesthesia. Together with controlled hypotension, these elements could have narrowed the safety margin such that a reflex vagal discharge from trigeminal stimulation precipitated profound bradyarrhythmia rather than self-limited bradycardia. A primarily drug-mediated cardioinhibitory event cannot be fully excluded; however, the timing with lateral nasal wall manipulation and the known literature on nasocardiac reflexes make a TCR-type mechanism the most plausible unifying explanation.

### 3.6. Management and Prevention Strategies

The cornerstones of TCR management are prompt recognition, cessation of the offending stimulus, and administration of anticholinergics. If bradycardia or asystole occurs, the surgeon should immediately stop manipulating the trigeminal nerve; this alone often restores sinus rhythm [26]. Atropine (0.01–0.02 mg/kg) or glycopyrrolate (0.005–0.01 mg/kg) can be given intravenously to block the vagal efferent [27]. In refractory cases epinephrine and standard ACLS may be required [28]. Continuous cardiovascular monitoring with capnography and arterial line ensures rapid detection of hemodynamic changes. Adequate depth of anesthesia and avoidance of hypoxemia or hypercarbia reduce reflex excitability [26].

Prevention strategies include the following:

1. Identification of high-risk moments—The anesthesia team should be alerted before pterygomaxillary disjunction, maxillary down-fracture, or the placement of retractors and bite blocks, which are high-risk moments for TCR [24]. Gentle, gradual manipulation rather than abrupt traction may reduce the reflex [29].
2. Pharmacologic prophylaxis—A mandibular nerve block (e.g., Gow-Gates) or local infiltration may attenuate afferent input and reduce TCR episodes during BSSO in selected patients but does not eliminate risk [23,30]. Prophylactic atropine or glycopyrrolate reduces TCR-related bradycardia in ocular surgery, yet the benefit in long orthognathic cases is uncertain [31].
3. Optimization of physiological parameters—Maintain oxygenation, normocapnia, and adequate anesthetic depth; avoid excessive opioids and β-blockers when feasible [4].
4. Team communication—Surgeons and anesthetists must coordinate to anticipate TCR and prepare to intervene quickly [7].

### 3.7. Literature Review of Reported Cases

*Search strategy.* For this mini review, we searched PubMed and Scopus up to September 2025 using the terms “trigeminocardiac reflex,” “trigeminovagal reflex,” “oculocardiac reflex,” “orthognathic,” “Le Fort,” “sagittal split,” “mandibular osteotomy,” “bradycardia,” and “asystole” and screened reference lists of relevant articles. We included reports describing orthognathic procedures with clearly documented intraoperative bradycardia or asystole temporally associated with trigeminal manipulation. For several Japanese case reports, we relied on the English-language summary provided by Hasegawa et al. (2025) [32], which we acknowledge as a limitation.

Table 2 summarizes key characteristics of published TCR episodes during orthognathic surgery. Each case involved mechanical stimulation of trigeminal nerve branches during Le Fort I osteotomy or BSSO. The table highlights the moment of reflex activation, and the measures taken to resolve it. While bradycardia-only cases are included for completeness, the asystolic/arrest cases are emphasized because they capture the maximal clinical severity and mirror the index event in the present report. Anticholinergics are often administered, and CPR may be required if asystole is prolonged.

**Clinical implications**

**Figure 1:**
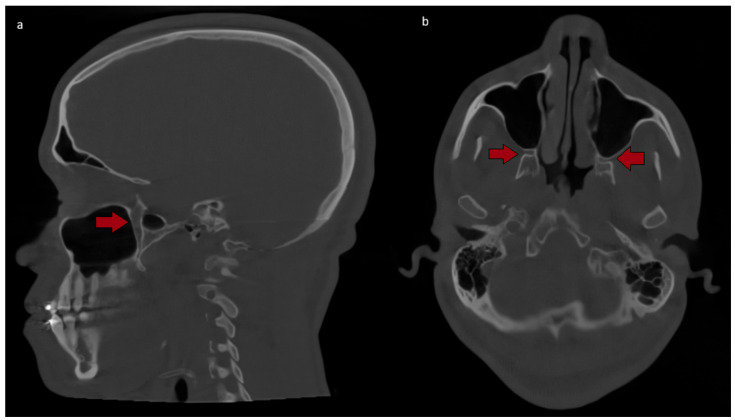
Preoperative cone-beam computed tomography (CBCT) of the maxilla illustrating the anatomic “danger zone” for V2-mediated trigeminocardiac reflex. (**a**) Sagittal view and (**b**) axial view at the level of the pterygomaxillary junction (solid arrow).

1. Anticipate TCR during Le Fort I osteotomy: Surgeons should be aware that lateral nasal osteotomy, pterygomaxillary disjunction. Anticipate TCR during Le Fort I osteotomy: Surgeons should be aware that lateral nasal osteotomy, pterygomaxillary disjunction and maxillary down-fracture may elicit TCR-type events, particularly in young male patients. Anticipatory communication with anesthesia team is vital. Preoperative CBCT (Figure 1) can help visualize the posterior maxillary wall, pterygomaxillary junction, and their proximity to the pterygopalatine fossa, reinforcing awareness of this high-risk anatomic zone during planning and execution of the osteotomies.
2. Monitor closely: Continuous heart rate and blood pressure monitoring allows prompt detection of bradycardia or asystole. Arterial lines are recommended for high-risk cases.
3. Prevent and prepare: Consider regional nerve blocks (e.g., mandibular nerve block) selectively—especially during BSSO—recognizing variable efficacy and limited data for routine prophylaxis.
4. Manage promptly: Stop the stimulus, administer atropine or glycopyrrolate, and be prepared to initiate CPR if asystole persists.
5. Refined surgical technique: Surgeons should prioritize gentle, controlled, and intermittent manipulation of tissues, with an immediate cessation of the surgical stimulus upon any physiological sign of the reflex.
6. Team-based approach: Fostering seamless communication and a shared understanding of TCR between surgeons and anesthesiologists is non-negotiable for ensuring a rapid and effective response to any event.
7. Inform the patient: Discuss the rare risk of TCR and potential intra-operative complications during informed consent.

### 3.8. Limitations

This case report has several important limitations. First, archived ECG tracings and anesthetic monitor screenshots from the event were unavailable, preventing us from demonstrating the second-by-second temporal relationship between surgical stimulus and hemodynamic collapse. Second, the duration of electrical asystole can only be inferred from anesthesia and code records, which document recognition of arrest, initiation of chest compressions, epinephrine administration, and return of spontaneous circulation within approximately one minute but do not provide exact onset and offset times. Third, we did not perform EEG or formal cognitive testing after the event; postoperative neurologic assessment relied on serial bedside examinations. Finally, our literature review is narrative and focused rather than fully systematic, and some orthognathic cases were extracted from secondary summaries, which may omit details.

## 4. Conclusions

The trigeminocardiac reflex is an uncommon but important cause of intraoperative bradycardia or asystole during orthognathic surgery. In our patient, a ventricular asystolic arrest occurred during lateral nasal osteotomy and is most consistent with a peripheral TCR-type event occurring in the setting of controlled hypotension and multimodal anesthesia. Rapid recognition, cessation of the stimulus, and appropriate resuscitation led to an excellent outcome. Orthognathic maneuvers predominantly risk peripheral TCR (V2/V3), particularly at the lateral nasal wall, down-fracture, pterygomaxillary disjunction, and mandibular manipulations. Surgeons should anticipate these moments, coordinate closely with anesthesia team, and be prepared to halt manipulation and administer vagolytics and advanced resuscitative measures when required. Further prospective data are needed to refine prevention strategies, characterize anesthetic and pharmacologic cofactors, and improve risk stratification.

## Figures and Tables

**Table 1:** Chronology of medications, surgical steps, and vitals surrounding intra-operative ventricular asystole during Le Fort I osteotomy.

Time	Anesthetic Events	Surgical Events	Vitals	Notes
07:41–08:15	OR arrival; induction (propofol 150 mg, fentanyl 100 µg, midazolam 2 mg, rocuronium 50 mg); nasotracheal intubation; antibiotic (ampicillin–sulbactam 3 g IV).	Prep/drape; circumvestibular incision; local anesthesia (2% lidocaine w/epinephrine) for bilateral IAN blocks and local infiltration.	Baseline HR 74; BP 146/97; SpO_2_ 100%.	—
08:15–09:10	Labetalol 5 mg IV; hydralazine 5 mg IV; IV fluids (LR).	Exposure; stepped Le Fort I osteotomies with reciprocating saw (right then left).	HR 80–90; intermittent hypertension (up to ~160/141); SpO_2_ 100%.	—
09:10–09:24	Hydralazine 5 mg IV; fentanyl 50 µg IV; rocuronium 20 mg IV.	Pterygomaxillary separation (Burton osteotome) and guarded left lateral nasal wall osteotomy (no down-fracture).	HR 74–83; BP 109/67–119/74; SpO_2_ 99–100%.	Index stimulation period.
09:25–09:26	Code start; epinephrine 1 mg IV; CPR.	All surgical maneuvers halted.	Asystole; SpO_2_ 99–100%; ROSC achieved in <1 min (HR ~37 at ROSC).	Suspected V2-mediated TCR.
09:31–11:20	Atropine 0.2 mg IV x2; ephedrine 10 mg IV; norepinephrine infusion (09:46–09:53); arterial line/IV access; transfer intubated to SICU.	Hemostasis and closure; Surgiflo/Avitene at pterygomaxillary junctions; throat pack removed; case aborted.	HR 48–67; BP nadir ~75/64 improving to 128/75; SpO_2_ 99–100%.	Down-fracture not completed.

ETT: endotracheal tube; IAN: inferior alveolar nerve; IV: intravenous; BP: blood pressure; HR: heart rate; ROSC: return of spontaneous circulation.

**Table 2:** Published TCR events in orthognathic surgery with trigger, rhythm, intervention, and outcome (including this case).

Year, Author	Sex	Age	Race	Surgery	Index Event	Manifestation	Management and Outcome
1989, Ragno et al. * [20]	M	17	Caucasian	LF 1	During down-fracture of the maxilla	Asystole (several seconds)	Surgery was stopped; atropine 0.4 mg; glycopyrrolate 0.3 mg; surgery completed uneventfully.
1990, Precious et al. * [33]	Six cases; details not provided			LF 1	Maxillary mobilization and advancement	Asystole or bradycardia (20–40 bpm)	Glycopyrrolate 0.2 mg; chest compression.
1991, Lang et al. (Cases #1) * [34]	F	28	Caucasian	LF 1	Pterygomaxillary osteotomy	Asystole	Surgery was stopped; atropine 0.6 mg.
1992, Lang et al. (Cases #2) * [34]	F	26	Caucasian	LF 1, BSSO, genioplasty	Placement of retractor during BSSO	Asystole	Surgery was stopped; inferior alveolar nerve block; atropine 0.6 mg; surgery completed uneventfully.
1993, Lang et al. (three cases) [34]	F	38	Caucasian	LF 1 and BSSO	Forward traction of the maxilla during Le Fort I	Bradycardia (95 to 65 bpm)	Surgery was stopped; atropine 1.2 mg; surgery completed uneventfully.
1994, Campbell * et al. [23]	F	35	Chinese	LF 1 and Hofer	Maxillary tuberosity osteotomy	Asystole (10 s)	Surgery was stopped; O2 100%; atropine 0.6 mg; surgery completed uneventfully.
2009, Sanuki et al. * [35]	F	31		BSSRO	Mandibular soft tissue dissection	Asystole (8 s)	Surgery was stopped; atropine 0.5 mg; local anesthesia; surgery completed uneventfully.
2012, Miyamoto et al. [36]	F	18		LF 1, BSSRO, genioplasty	Suturing the mandibular mucoperiosteal flap	Bradycardia	Surgery was stopped; atropine; cardiac massage; surgery completed uneventfully.
2013, Wakasugi et al. * [37]	M	21		LF 1, BSSRO	Manipulation of the mandible	Asystole	Interruption of the surgery.
2013, Kumasaka et al. [38]	F	18		LF 1, BSSRO	Placing a retractor along the medial aspect of the mandibular ramus	Bradycardia, hypotension	Interruption of the surgery; anticholinergic drugs.
2013, Kumasaka et al. [38]	M	39		BSSRO	Placing a retractor along the medial aspect of the mandibular ramus	Bradycardia	Interruption of the surgery.
2019, Baronos et al. * [22]	M	26	Asian	LF 1, BSSO, genioplasty	Placement of bite block towards end of surgery	Asystole (10 s); severe bradycardia (30–40 bpm) with repeat placement of bite block	Surgery was stopped; glycopyrrolate 0.4 mg.
2019, Kim et al. [39]	M	23		BSSO	Miniplate fixation during BSSO	Bradycardia (HR 25–30 bpm)	Surgery was stopped; lidocaine (80 mg) and glycopyrrolate (0.2 mg); surgery completed uneventfully.
2020, Sugiyama et al. [40]	F	31		LF 1 and BSSO	Splitting of the mandibular ramus	Bradycardia (29 bpm)	Surgery was stopped; atropine 0.5 mg; local anesthesia; surgery completed uneventfully.
2020, Maharaj et al. [21]	M	45		LF 1 and BSSO	Mobilization of the maxilla with Rowe’s dis-impaction forceps	Bradycardia	Surgery was stopped; surgery completed uneventfully.
2024, Alshalawi et al. [41]	M	32	Saudi	LF 1, BSSO, genioplasty	Down-fracture of the maxilla	Bradycardia (25 bpm)	Surgery was stopped; atropine 0.5 mg ×2; surgery completed uneventfully.
2024, Ortiz-Peces et al. [7]	M	36	Caucasian	LF 1 and BSSO	Mandibular nerve disjunction in BSSO; pterygomaxillary disjunction	Bradycardia (35 bpm); asystole (5 s)	Surgery was stopped; atropine 0.5 mg; surgery completed uneventfully.
2025, Hasegawa et al. (Case 1) [32]	F	39		LF 1 and BSSRO	Down-fracture of the maxilla	Bradycardia (46 bpm)	Surgery was stopped; supplemental local anesthesia; surgery completed uneventfully.
2025, Hasegawa et al. (Case 2) [32]	M	26		LF 1 and BSSRO	Splitting of the left mandibular ramus while autologous blood transfusion was being given	Bradycardia (52 bpm); hypotension (54/25 mmHg)	Autotransfusion was paused; 4 mg ephedrine was administered; surgery completed uneventfully.
2025, Current case *	M	32	African American	LF 1	Lateral nasal osteotomy	Asystolic arrest; code and ROSC within <1 min (exact asystole duration unknown)	Surgery was stopped; epinephrine 1 mg; chest compression; surgery abandoned.

The original case reports by Wakasugi et al. (2013) [37] and Kumasaka & Miura (2013) [38] were not directly accessible; the metadata and case details were obtained from the article by Hasegawa et al. (2025) [32]. BSSO: bilateral sagittal split osteotomy; BSSRO: bilateral sagittal split ramus osteotomy; LF 1: Le Fort 1 osteotomy; bpm: beats per minute. * Cases with asystole.

## Data Availability

No new data were created or analyzed in this study. Data sharing is not applicable to this article.
